# Psychometric properties of the International Society of Wheelchair Professionals’ basic manual wheelchair-service-provision knowledge Test Version 1 and development of Version 2

**DOI:** 10.1371/journal.pone.0281584

**Published:** 2023-03-23

**Authors:** Yohali Burrola-Mendez, R. Lee Kirby, Paula W. Rushton, Silvana Contepomi, Amira E. Tawashy, Padmaja Kankipati, Norma Jiménez García, Lauren Terhorst, Mary Goldberg, Jon Pearlman

**Affiliations:** 1 School of Rehabilitation, Université de Montréal, Montréal, Canada; 2 CHU Sainte-Justine Research Centre, Montréal, Canada; 3 Division of Physical Medicine and Rehabilitation, Dalhousie University, Halifax, Canada; 4 Argentine Assistive Technology Association, Buenos Aires, Argentina; 5 School of Occupational Therapy, Dalhousie University, Halifax, Canada; 6 Assistive Technology and Rehabilitation Consultant, Bangalore, India; 7 Assistive Technology Consultant, Mexico City, Mexico, United States of America; 8 Department of Occupational Therapy, SHRS Data Center, University of Pittsburgh, Pittsburgh, Pennsylvania, United States of America; 9 Department of Rehabilitation Science and Technology, University of Pittsburgh, Pittsburgh, Pennsylvania, United States of America; Faculty of Associated Medical Sciences, Chiang Mai University, THAILAND

## Abstract

**Introduction:**

Valid and reliable scores from measurement tools to test competency in basic manual wheelchair-service-provision are needed to promote good practice and support capacity building. The International Society of Wheelchair Professionals’ (ISWP) Basic Test Version 1 in English, launched in 2015, is the most frequently used outcome measure tool to test basic manual wheelchair-service-provision knowledge and is part of an international certification process. Despite the wide acceptance and use of the test, its psychometric properties have not yet been established. The objectives of this study were 1) to evaluate the test’s psychometric properties, 2) to develop the test’s Version 2, and 3) to evaluate the content validity of the new version.

**Methods:**

For Objective 1, methods from the Classical Test Theory were used to obtain items’ difficulty, item discrimination index and domains’ reliability. For Objective 2, a team of experts in wheelchair service delivery and education conducted a systematic qualitative review of the questions’ text and answers and updated them using evidence-based guidelines. For Objective 3, an external team reviewed the clarity, relevance and domain allocation of the developed items using a 4-point Likert scale. Descriptive statistics were used to describe and characterize the results for each objective. Item-content (I-CVI) and Scale-content (S-CVI) validity indexes were calculated to compute content validity.

**Results:**

For Objective 1, all domains in the test were below the threshold for acceptable internal consistency reliability; 80% of the total test pool (116 items from the total pool of 145) did not meet the thresholds for item difficulty and index of discrimination suggested in the literature. Of the items in the Test, 78% could be responded to intuitively and 66% did not distinguish between test-takers who were knowledgeable in the content area and those who were not. For Objective 2, experts found concerns such as items being grouped in the wrong domain, being repeated, not using person-first language, and using terms inconsistently. Thirty-four (23.4%) items were dropped and 111 (76.5%) were updated. In addition, 61 new items were developed. Members re-categorized the items and proposed a new classification of subdomains. For Objective 3, good agreement between subject-matter experts was found; the S-CVI calculated using the I-CVIs related to item clarity was 84% while using the I-CVIs related to item relevance was 98%. Only 7 items (4.1%) were deemed to be in the wrong domain and 4 items (2.3%) were considered irrelevant and dropped.

**Conclusion:**

The psychometric evidence in support of ISWP Basic Test Version 1 in English is suboptimal. A new set of items developed by experts in the field has shown excellent content validity. Ongoing assessments will be needed as ISWP Basic Test Version 2 is implemented and monitored.

## Introduction

Capacity building in wheelchair-service-provision is a key component in promoting good practice and developing sustainable wheelchair provision systems worldwide [1]. To support competency development among wheelchair service personnel, valid evidence-based and reliable measurement tools are needed. A recent scoping review on wheelchair-service-provision education highlighted the availability and use of open-source training packages that have been supporting the development of competencies in wheelchair-service-provision [2]. In particular, the World Health Organization Wheelchair Service Training Package-Basic Level (WHO Basic Package) [3] (a training resource that supports wheelchair service delivery for people not requiring postural support) and the Wheelchair Skills Program (WSP) [4] (protocol related to the assessment and training of wheelchair skills) were the two most frequently reported training programs used to improve competency in wheelchair-service-provision among rehabilitation students, clinicians and lay health workers [2].

The evaluation of basic manual wheelchair-service-provision knowledge was the most frequently evaluated competency, and the International Society of Wheelchair Professionals (ISWP) Basic Manual Wheelchair-Service-Provision Knowledge Test Version 1 (ISWP Basic Test Version 1) was the most frequently used outcome measure [2, 5]. Basic manual wheelchair-service-provision knowledge is needed to provide wheelchair services to people with mobility impairment who can sit upright without additional postural support [3]. Studies that have used the ISWP Basic Test Version 1 targeted rehabilitation students and clinicians in Colombia [6–8], India [9], Mexico [9], and the United States [10]. The ISWP Basic Test Version 1 was used to measure changes in manual wheelchair-service-provision knowledge after a training intervention [6, 9, 10] or manual wheelchair-service-provision knowledge among rehabilitation students [7, 8]. Furthermore, a recent exploratory analysis revealed associations between the ISWP Basic Test Version 1 and test takers’ education, motivation, and country income setting [11].

The ISWP Basic Test Version 1, launched in 2015, is an online multiple-choice test that was developed based exclusively on the WHO Basic Package [3]. The test includes 75 items taken from a total pool of 145 items that are organized into 7 domains of wheelchair-service-delivery knowledge: *Assessment*, *Prescription*, *Fitting*, *Production*, *User training*, *Process* and *Maintenance and repairs* [5]. The domains are aligned with the content of the WHO 8-steps of wheelchair service delivery [3, 12] but they were grouped differently. In the ISWP Basic Test Version 1, the domain *Process* consists of *Referral and appointment* and *Funding and ordering* [5]. The domains have different weights based on the pre-set number of questions within each domain [5]. To reduce the likelihood of receiving the same question when taking the test multiple times, each domain has a pool of questions from which only a subset is drawn. The ISWP Basic Test Version 1 settings include: 1) random distribution of questions from each domain’s pool of questions, 2) forced completion, requiring participants to complete the test in a one-time entry; and 3) immediate scoring of the test with the opportunity to review both correct and incorrect answers [5]. A total percentage of test scores of at least 70% is considered a passing grade.

As of November 2022, the ISWP Basic Test Version 1 has been taken by 6,619 test takers from 106 countries and is part of an international wheelchair-service-provision fee-based certification process (https://wheelchairnetwork.org/resource-library/iswp-tests/) that has been pursued by 367 people. The ISWP Basic Test Version 1 has been translated into 14 languages. The languages are requested by the community and undergo a formal forward-only translation process that includes a review by two bilingual subject matter experts that are specifically recruited for this task [13]. Recently, Rushton et. al [14] conducted a translation and adaptation of the ISWP Basic Test Version 1 from English to French-Canadian along with a preliminary evaluation of its internal consistency. Their results indicated a low degree of internal consistency between the items in the ISWP Basic Test and the translated French-Canadian version. The authors recommended evaluating the internal consistency of the ISWP Basic Test.

Despite the rapid uptake and use of the ISWP Basic Test Version 1, its psychometric properties have not been reported and the test has not been revised nor updated since its launch. The ISWP Basic Test lacks evidence of its content validity and reliability (e.g., internal consistency) to support the test’s relevance for its intended purpose. The methods followed to develop the ISWP Basic Test have multiple limitations such as the exclusion of relevant evidence-based materials (e.g., the WSP) the lack of methodological details related to the development of items, selection of domains and assessment of the clarity and relevance of questions [5]. To enhance the quality of research and to ensure that a valid and reliable test is being used as part of an international wheelchair service provision certification process, the ISWP Basic Test Version 1 needs to be revised, evaluated, and updated considering its items’ performance and the inclusion of other relevant training packages.

The three objectives of this project were: 1) to evaluate the psychometric properties (i.e., item difficulty, item discrimination index, and domains’ internal consistency reliability) of the ISWP Basic Test Version 1; 2) to develop the ISWP Basic Test Version 2; and 3) to evaluate the content validity (i.e., item- and scale-content validity indices [I-CVI and S-CVI respectively]) of the items included in ISWP Basic Test Version 2. This study aims to improve a measurement tool to test wheelchair-service-provision competency and contribute to supporting good practice and capacity building.

## Methods

### Objective one: Evaluate the psychometric properties of the ISWP Basic Test

We analyzed the data from the ISWP Basic Test Version 1 in English hosted on Test.com from January 1st, 2015, to January 24, 2020. The inclusion criteria for the datasets considered were successful test attempts (i.e., test-takers who completed both the demographic and the multiple-choice sections), and first attempts by test-takers. All test-takers gave informed consent to share their test-scores and sociodemographic information for the purpose of this study. The study was approved by the University of Pittsburgh’s Institutional Review Board (STUDY19100169).

### Statistical approach

#### A) Investigate passing rates and possible relations between test-takers characteristics and total test scores

The total test scores were analyzed for normality of distribution using the Shapiro-Wilk test (W) and visually inspected for normal-distribution assumption using a histogram. Descriptive statistics, measures of central tendency, and measures of spread were calculated to summarize and describe the data and to determine the percentage of test-takers who passed the test. Significance was set at α = 0.05. All analyses were performed using STATA software, version 15.

#### B) Investigate questions (items) and domains’ performance

Methods from Classical Test Theory were used to evaluate the data. The following item-level statistics were obtained:

- Item difficulty (p). This was the proportion of test-takers who answered an item correctly [15, 16].
$$p_{j}=\frac{Number\;of\;people\;who\;correctly\;answer\;item_{j}}{N}$$
  This proportion was obtained by dividing the number of test-takers who responded correctly to an item (j) by the total number of respondents (N). The p-values have a range from 0.00 to 1.00. Lower p-values are indicative of more difficult items while higher p-values suggest easier items [15]. Multiple-choice questions are affected by random guessing, the number of answer options and partial knowledge that test-takers may have and allow them to eliminate answer options before guessing [16]. To adjust for random guessing, acceptable p-values for items with 4-answer options are ≤ 0.74 [16, 17].
- Item discrimination index (IDI). This index allows differentiating between test-takers who know the criterion of interest and those who do not [15, 16]. IDIs were obtained by organizing the group’s total test scores in descending order and then grouping the upper and lower 27% [18]. After the groups had been identified, the IDI was obtained:
$$IDI=p_{u}-p_{l}$$
  *P*_*u*_ is the proportion in the upper group who answer the item correctly and *p*_*l*_ is the proportion in the lower group who answer correctly. IDIs have a range from -1.00 to 1.00. Positive values indicate that the value discriminates in favor of the upper group, and negative values are in favor of the lower-scoring group, indicating it is a reverse discriminator [16]. As suggested by the literature, IDIs <0.30 were flagged for revision as these items are marginal and should be eliminated or completely revised [19].
- Domains’ reliability. We used the Kuder-Richardson Formula 20 (KR-20) to estimate internal consistency. KR-20 is commonly used to test the internal consistency of an achievement test and the test measures the unidimensional trait [20–22].
$$r_{KR20}=\left(\frac{k}{k-1}\right)\left(1-\frac{\sum pq}{\sigma^{2}}\right)$$
- rKR20 is the Kuder-Richardson formula 20
- k is the total number of test items
- Σ indicates to sum
- p is the proportion of the test-takers who pass an item
- q is the proportion of test-takers who fail an item
- σ2 is the variation of the entire test
- To meet the unidimensional condition, KR-20 coefficient was calculated and analyzed for each domain separately. KR-20 coefficients range from 0.0 to 1.0 As per the literature, a KR-20 value ≥0.80 is considered an acceptable level of internal consistency reliability [22].

### Objective two: Develop the ISWP Basic Test Version 2

#### Committee formation

A purposive sampling method was used to recruit an international group of stakeholders to form the Assessment Tools Committee (ATC). ISWP sent 10 invitation letters describing the scope of the project, time commitment, and work effort. The inclusion criteria were at least 5 years of experience in wheelchair service delivery, familiarity with the WHO Basic Package and the WSP, being actively engaged in the wheelchair sector, have access to a computer with Internet connectivity and be fluent in English. Potential members who agreed to be part of the committee responded to a demographic survey that recorded socio-demographic characteristics; perceived familiarity with the content of the WHO and WSP materials; and perceived confidence in interpreting components of psychometric analysis, correlations, and research methodology. The purpose of the ATC was to conduct a qualitative review of the items in the ISWP Basic Test guided by the quantitative analysis from Objective 1.

#### Revision process

Before the start of the revision process, members of the ATC received training on the use of the assessment forms, the methodology for the revision of the items, and the evidence-based guidelines for the development and improvement of items. The training lasted two hours, was online, synchronous, and facilitated by the project lead (YBM) using Zoom (https://zoom.us/). The project lead was a physiotherapist and rehabilitation researcher with seven years of experience designing and implementing educational interventions related to improving wheelchair service provision competencies among rehabilitation professionals. After the ATC training, an online repository with relevant materials (i.e., articles, books, videos, and presentations) was made available for all ATC members. The WHO Basic Package [3] and the WSP [4] were the training packages used to review, update, and create new items. ATC members were encouraged to create new items when not all aspects of basic manual wheelchair-service-provision knowledge were covered by the current items. Seven teams, each consisting of 2–4 members of the ATC, were formed; each team was assigned to review one domain of the ISWP Basic Test (i.e., *Assessment*, *Prescription*, *Fitting*, *Production*, *Users’ training*, *Process*, *and Maintenance and repairs*). Members of the ATC were purposefully assigned to the teams considering their area of expertise. In addition, a senior member (RLK) and the project lead (YBM) participated in all groups.

The revision process consisted of four phases: Phase 1: Analysis and revision. This phase included two sequential steps.

1. *Qualitative revision of the questions’ text and answers*. ATC members evaluated the quality of the questions using the Question Guidelines Table (S1 Table), a matrix created by the research team that included evidence-based guidelines for developing questions’ text and answers and prompted ATC members to determine if the questions met or not the criteria.
2. *Proposed changes to the question texts and answers*. ATC members used the Domain Feedback Form (S2 Table) to analyze the results from the quantitative analysis (from Step 1) and propose changes. The Domain Feedback Form consisted of three sections:
  - Section 1. Domain summary table. This table was informative and included the total number of items in the domain, the total number of items flagged for revision, and the domain’s internal consistency reliability.
  - Section 2 & 3. Flagged and unflagged questions for revision. These sections grouped the questions that did and did not meet the thresholds established by the methods from Classical Test Theory (Objective 1). The tables included question texts and answers, p-values, IDIs, and frequency and percentage of answer-options selections. An open section next to each question prompted ATC members to propose changes. ATC members were encouraged to use the Question Guidelines Table previously completed to address identified issues and to propose changes that included evidence-based guidelines. ATC members were asked to submit the Domains Feedback Form prior to Phase 2.

Phase 2: Online Team Synchronous Meetings. During the online teams’ meetings, ATC members discussed the results from Phase 1 and the proposed changes until consensus was reached. When new items were created, members rated them using the Question Guidelines Table to ensure the new questions met evidence-based guidelines. Consensus was considered to have been reached when the majority of ATC members approved changes to existing items and the inclusion of new items. Once consensus was reached, a domain’s pre-final draft with all items was created. The meetings were facilitated by the project lead on the Zoom platform, they were recorded and made available for members who were unable to attend.

- Phase 3: Pre-final version. The domains’ pre-final drafts were distributed to all ATC members for their review. If ATC members disagreed with any item, they emailed their concerns to the project lead and met with the corresponding team that created the item. When consensus was reached, the pool of questions was finalized and considered ready for the last phase.
- Phase 4: Classification and final revision. The project lead and a senior member categorized all questions independently and proposed the domains’ definitions using the WHO Basic Package and the WSP content as references. The new domains with their definitions and questions were distributed to all ATC members for their review and approval. When a consensus was reached, the final set of questions was ready for content validity analysis.

### Objective three: Evaluate the content validity of the items included in ISWP Basic Test Version 2

#### Participant recruitment

A purposive sampling method was used to recruit at least 8 participants comprising researchers, educators and clinicians to form the Subject Matter Experts Group (SMEG). This sample size aligned with standard recommendations [23, 24]. To be eligible to participate, experts had to have had at least 5 years of experience in wheelchair service delivery, be familiar with the WHO Basic Package and the WSP, be actively engaged in the wheelchair sector, have access to a computer with Internet connectivity, be fluent in English, and not be members of the ATC. Completing the Qualtrics survey served as participants’ consent to participate in the study.

#### Survey development and administration

To evaluate content validity, a survey was created consisting of a socio-demographic questionnaire and the item pool of questions developed by the ATC. The survey asked participants to rate the clarity and relevance of each item on a 4-point Likert scale (1 = strongly disagree to 4 = strongly agree), provide open-ended feedback regarding content and wording immediately after rating each item and indicate if the item was representative of the allocated domain. The use of a 4-point Likert scale has been suggested in the literature to avoid a neutral midpoint [23]. Ratings were dichotomized as “agree” (for those who responded 3 [6] or 4 [6]) and “disagree” (for those who responded 2 [6] or 1 [6]), following the typical recommendation for computing content validity [24, 25]. The survey was hosted in Qualtrics^®^, a web-based survey tool, and distributed online via an external link.

Members of the SMEG attended a one-hour synchronous meeting in which the scope of the project, instructions, and structure of the survey were explained. The survey remained open for four weeks and allowed participants to save their progress and complete the survey in multiple attempts. Participants who completed the survey received a letter from ISWP acknowledging their time and contributions, and they are listed in the Acknowledgments section of this paper.

#### Content validity assessment

Descriptive statistics were used to describe and characterize the sample. The I-CVI and the S-CVI were calculated from the item pool. As recommended in the literature, the I-CVI was calculated by taking the number of participants in agreement based on the dichotomized rating scale and dividing it by the total number of participants in the SMEG who answer the question [23, 24]. The S-CVI was calculated by summing the I-CVIs and dividing it by the total number of items; this method is referred to in the literature as S-CVI/Average [23, 24]. Acceptable agreement for the I-CVI was set as ≥ 0.78 and for S-CVI at ≥ 0.90 [23, 24, 26, 27]. The ATC used the I-CVIs and the open-ended responses from the survey to guide them in revising, updating, or deleting the items.

## Results

### Objective one: Evaluate the psychometric properties of the ISWP Basic Test Version 1

#### Investigate passing rates and possible relations between test-takers characteristics and total test scores

Table 1 includes the characteristics of the population, all total test-takers, and test-takers who passed and did not pass the test. A total of 1276 test attempts of the ISWP Basic Test Version 1 in English were completed between January 1, 2015, to January 24, 2020; 1108 (86.8%) were successful (i.e., completed both the demographic and multiple-choice sections) and represent 947 unique users on their first test attempts. Four users were removed from the analysis due to incomplete attempts that were not captured in the first exclusion. The sample size retained for analysis consisted of 943 test-takers, a near gender-balanced representation with a mean age of 35 years. Test-takers were geographically distributed in five continents (i.e., Asia, Africa, America, and Europe) with the majority located in Asia. The most frequent educational level was a bachelor’s degree and Physical Therapist was the most frequently reported certification. More than half of the test-takers had less than 3 years of experience in wheelchair-service-provision by the time they took the test and most test-takers reported spending 3–20 hours per week in wheelchair service delivery. About three-quarters of the test-takers passed the ISWP Basic Test in their first attempt; in this group, the highest domain mean score was from *Process* and the lowest was from *Fitting*. About one-quarter of test-takers failed the ISWP Basic Test; in this group, the domain with the highest score was *Assessment* and the lowest was *Fitting*.

**Table 1:** Characteristics of test-takers and test scores of ISWP Basic Test Version 1.

Characteristics	Total test-takers (n = 943)	Pass (n = 715)	Fail (n = 228)
**Gender, n (%)**			
Men	497 (52.7)	361 (50.5)	136 (59.7)
Women	446 (47.3)	354 (49.5)	92 (40.4)
Age, mean (SD)* **[SEM]**^ϕ^	34.5 (10.9)^μ^	34.6 [0.41]	34.1 [0.7]
**English spoken at home, n (%)**	315 (31.3)^€^	263 (36.8)	52 (22.8)
**Regions, n (%)** ^α^			
Asia	510 (54.3)	370 (52)	140 (61.4)
Africa	168 (17.9)	112 (15.7)	56 (24.6)
North America	163 (17.3)	147 (20.7)	16 (7)
Oceania	38 (4)	35 (4.9)	3 (1.3)
Europe	35 (3.7)	29 (4.1)	6 (2.6)
Latin America	26 (2.8)	19 (2.7)	7 (3.1)
**Educational level, n (%)**			
High School	59 (6.3)	34 (4.8)	25 (11)
Some College	147 (15.6)	95 (13.3)	52 (22.8)
2-year degree/associates degree	111 (11.8)	74 (10.4)	37 (16.2)
4-year degree/bachelor’s degree	380 (40.3)	309 (43.2)	71 (31.1)
Graduate degree/masters’ level	213 (22.6)	173 (24.2)	40 (17.5)
Graduate degree/MD, PhD	33 (3.5)	30 (4.2)	3 (1.3)
**Last year of formal training, n (%)**			
Still attending	210 (22.3)	171 (23.9)	39 (17.1)
Less than 4 years	344 (36.5)	257 (35.9)	87 (38.2)
5–8 years	134 (14.2)	97 (13.6)	37 (16.2)
More than 8 years	255 (27)	190 (26.6)	65 (28.5)
**Previous wheelchair training, n (%)**			
No	305 (32.2)	211 (29.5)	94 (41.2)
Yes	638 (67.7)	504 (70.5)	134 (58.8)
**Certification, n (%)**			
Physical therapist	201 (39.3)	169 (40.8)	32 (33)
Occupational therapist	144 (28.2)	113 (27.3)	31 (32)
Prosthetics and orthotics	76 (14.9)	59 (14.3)	17 (17.5)
WHO WSTP-B	64 (12.5)	48 (11.6)	16 (16.5)
WHO WSTP-I^β^	4 (0.8)	3 (0.7)	1 (1)
ATP^λ^	22 (4.3)	22 (5.3)	0 (0)
**Employment status, n (%)**			
Unemployed	202 (21.4)	150 (21)	52 (22.9)
Part-time 20hrs/week or less	125 (13.3)	100 (14)	25 (11)
Full time	616 (65.3)	465 (65)	151 (66.2)
**Work setting: yes, n (%)**			
Academic	344 (26.8)	270 (27.4)	74 (25)
In-patient	457 (35.6)	343 (34.8)	114 (38.5)
Outpatient	434 (33.8)	343 (34.8)	91 (30.7)
Other	48 (3.7)	31 (3.1)	17 (5.7)
**Age group served: yes, n (%)**			
Early childhood	475 (22.5)	378 (22.5)	97 (22.6)
Adolescents	478 (22.7)	389 (23.1)	89 (20.8)
Adults	714 (33.8)	551 (32.8)	163 (38)
Older adults	443 (21)	363 (21.6)	80 (18.7)
**Motivation for training: yes, n (%)**			
Professional growth	762 (61.6)	604 (62.9)	158 (56.8)
Personal growth	253 (20.4)	204 (16.5)	49 (4)
Required by academic program	137 (11.1)	96 (7.8)	41 (3.3)
Required by employer	86 (7)	56 (4.5)	30 (2.4)
**Wheelchair-service-provision experience, years, n (%)**			
<3 years	571 (60.6)	420 (58.7)	151 (66.2)
4–7 years	169 (17.9)	133 (18.6)	36 (15.8)
8 years or more	203 (21.5)	162 (22.7)	41 (18)
**Wheelchair-service-provision, hours, n (%)**			
Less than 3 hours/week	332 (35.2)	265 (37.1)	67 (29.4)
3–20 hours/week	431 (45.7)	309 (43.2)	122 (53.5)
More than 20 hours/week	180 (19.1)	141 (19.7)	39 (17.1)
**Member of an organization, n (%)**			
No	275 (29.2)	196 (27.4)	79 (34.7)
Yes	668 (70.8)	519 (72.6)	149 (65.4)
**Mean Scale scores, mean, %**^π^ **(SD) [SEM]**			
Total Wheelchair Service Basic Test (75)^¥^	56.6, 75.3% (9.4)	60.6, 80.8% [0.2]	43.6, 58.1% [0.6]
Assessment (19) ^¥^	15.6, 82.3% (2.5)	16.5, 86.9% [0.1]	12.8, 67.6% [0.2]
Prescription (12) ^¥^	8.9, 74.4% (1.9)	9.5, 79.5% [0.1]	7, 58.3% [0.1]
Fitting (10) ^¥^	5.6, 56% (1.9)	6.2, 61.5% [0.1]	3.9, 39.1% [0.1]
Production (5) ^¥^	3.6, 72% (1.2)	3.9, 78% [0.04]	2.7, 54% [0.1]
User’s training (15) ^¥^	11.25, 75% (2.5)	12.12, 80.8% [0.1]	8.53, 56.9% [0.2]
Process (10) ^¥^	8.4, 84% (1.9)	9.03, 90.3% [0.04]	6.5, 65% [0.2]
Follow up and maintenance (4) ^¥^	3.02, 75.5% (1)	3.3, 82.5% [0.03]	2.16, 54% [0.1]

*SD: Standard deviation

^ϕ^ SEM: Standard error of the mean

^μ^N = 938, 5 participants did not respond

^€^ Participants may enter multiple languages.

^α^ The countries that composed the regions were the following. Asia: Bhutan, Cambodia, China, Hong Kong, India, Indonesia, Iraq, Israel, Japan, Jordan, Lebanon, Malaysia, Myanmar, Nepal, Pakistan, Palestine, Philippines, Saudia Arabia, Sri Lanka, Tajikistan, Thailand, United Arab Emirates, and Yemen. Africa: Ethiopia, Ghana, Kenya, Malawi, Nigeria, Sierra Leone, South Africa, Sudan, Tanzania, Uganda, and Zimbabwe. North America: Canada and the United States of America. Oceania: Australia, New Zealand, and Papua New Guinea. Europe: Belgium, Czech Republic, Finland, Hungary, Italy, Norway, Romania, Spain, Switzerland, and the United Kingdom. Latin America: Argentina, Brazil, Colombia, El Salvador, Guatemala, Haiti, Mexico, Nicaragua, and Peru.

^β^ WHO WSTP-I: World Health Organization Wheelchair Service Training Package Intermediate level.

^λ^ ATP: Assistive Technology Professional.

^**π**^ The percentage was calculated by taking the mean score of each domain, multiplying it by 100, and then diving the result by the number of questions in that domain (or the total number of questions included in the test). Example, Assessment, mean (15.63 * 100 = 1563/19 = 82.3%).

^¥^ The numbers inside the parathesis indicate the total number of points/questions each domain has in a test. For instance, tests have 19 questions/points of Assessment.

#### Investigate domains internal consistency reliability and questions performance

None of the domains comprised in the ISWP Basic Test had a KR-20 coefficient ≥ 0.80 (Table 2) indicating a weak relationship between items on each domain. Of the 145 multiple-choice questions that comprised the ISWP Basic Test’s total pool, a total of 116 questions (80%) were flagged for revision as they did not meet the minimum thresholds for difficulty (i.e., p-values ≤ 0.74) and index of index of discrimination (i.e., values >0.30). From the flagged questions, 90 questions (77.6%) had a p-value >0.74 with indicates questions are extremely easy; 76 questions had an IDI < 0.30 which implies those questions are not discriminating between test-takers who know the content and those who do not. Fifty-seven questions (49.1%) did not meet both criteria. The domains with the highest percentage of questions flagged for revision were *Process* (92%), *Assessment* (91.2%), and *Prescription* (88.2%). In contrast, the domain with the lowest percentage of questions flagged for revision was *Production* (50%). Only 29 questions (20%) from the total pool of ISWP Basic Test questions met the criteria (i.e., p-value and IDI) and were not flagged for revision. Table 2 presents the domains’ internal consistency reliability and the questions’ performance and their item-level statistics.

**Table 2:** Domains’ internal consistency reliability and questions’ performance.

Domain	KR-20	N	Not flagged, n (%)	Flagged for revision, n (%)	p>0.74, n (%)	IDI < 0.30, n (%)	P>0.74 & IDI < 0.30, n (%)
Assessment	0.28	34	3 (8.8)	31 (91.2)	26 (76.5)	20 (58.8)	22 (64.7)
Prescription	0.31	17	2 (11.8)	15 (88.2)	11 (64.7)	11 (64.7)	7 (41.2)
Fitting	-0.26	22	7 (31.8)	15 (68.2)	6 (27.3)	12 (54.5)	3 (13.6)
Production	-0.69	12	6 (50)	6 (50)	4 (33.3)	4 (33.3)	2 (16.7)
Training	0.36	26	7 (26.9)	19 (73.1)	15 (57.7)	13 (50)	9 (34.6)
Process	0.04	25	2 (8)	23 (92)	22 (88)	14 (56)	13 (52)
Follow-up	-0.73	9	2 (22.2)	7 (77.8)	6 (66.7)	2 (22.2)	1 (11.1)
**Total, n (%)**		145	29 (20)	116 (80)	90 (77.6)	76 (65.5)	57 (49.1)

### Objective two: Develop the ISWP Basic Test Version 2

#### Committee formation

All ten recruited stakeholders accepted our invitation to join the ATC. Table 3 shows their characteristics. The group was represented mainly by females with a mean age of about 45 years and an average of 16 years involved in the wheelchair sector. Most members had completed a graduate degree. Three members (from the Philippines, the USA and Colombia) dropped out due to the workload.

**Table 3:** Assessment tool committee demographics.

Characteristics	Assessment Tool Committee (n = 10)
**Gender, Female, n (%)**	
Female	9 (90)
Male	1 (10)
**Age, mean (SD)** *	44.5 (12.45)
**Country of origin, n (%)**	
Argentina	1 (10)
Canada	3 (30)
Colombia	1 (10)
India	1 (10)
Mexico	2 (20)
Philippines	1 (10)
United States of America	1 (10)
**Primary language, n, (%)** ^μ^	
English	5 (45.45)
Spanish	4 (36.36)
Tagalog	1 (9.09)
Tegulu	1 (9.09)
**Secondary language, n (%)**	
English	4 (40)
French	2 (20)
Hindi	1 (10)
Italian	1 (10)
Kannada	1 (10)
Spanish	1 (10)
**Educational level, n (%)**	
4-year degree/bachelor’s degree	1 (10)
Graduate degree/masters’ level	4 (10)
Graduate degree/MD, PhD	5 (50)
**Profession, n (%)**	
Biomedical Engineer	2 (20)
Educator	1 (10)
Occupational Therapist	3 (30)
Physical Therapist	3 (30)
Physician	1 (10)
**Primary Occupation, n (%)**	
Consultant	4 (40)
Professor/Researcher	4 (40)
Service Provider	1 (10)
Student	1 (10)
**Years involved in wheelchair sector, n (SD)**	16.1 (10.74)
**Wheelchair service providers, n (%)**	7 (70)
Years providing wheelchair services	13.86 (13.87)
**Have taken the ISWP Basic Test: yes, n (%)**	7 (70)

* SD: Standard deviation

^μ^: N = 11, one participant entered 2 primary languages

### Revision process

#### Phase 1: Analysis and revision

*Qualitative revision of the question texts and answers*. Table 4 presents the average percentage of questions by sub-domain that met each criterion. Overall, the lowest average percentage of agreement across domains in the stem guidelines were ‘*stem is meaningful by itself and presents a definite problem’* and ‘*stem does not contain irrelevant material’* while from the answer options’ guidelines were ‘*alternatives are free from clues’* and ‘*alternatives are mutually exclusive’*.

**Table 4:** Average percentage of questions that met each criterion.

Guidelines	Assessment	Prescription	Fitting	Production	User’s Training	Process	Follow-up & Maintenance
**Reviewers, n** *	**3**	**4**	**4**	**3**	**4**	**3**	**3**
**Sample, n** ^μ^	**34**	**17**	**22**	**12**	**26**	**25**	**9**
**Stem criteria**							
Stem is meaningful by itself and presents a definite problem.	87%	90%	68%	67%	83%	77%	89%
Stem does not contain irrelevant material.	90%	78%	77%	81%	81%	88%	70%
Stem is a question or partial sentence.	95%	91%	98%	94%	98%	98%	100%
Stem is negatively stated (italics/CAPS) only when significant learning outcomes require it.	83%	N/A^¥^	N/A	N/A	N/A	N/A	N/A
Stem targets a specific cognitive process.	100%	94%	90%	86%	96%	90%	93%
**Answer criteria**							
All alternatives are plausible.	71%	79%	85%	61%	86%	81%	93%
Alternatives are stated clearly and concisely.	81%	81%	75%	67%	77%	79%	81%
Alternatives are mutually exclusive.	72%	72%	67%	75%	78%	71%	89%
Alternatives are homogenous in content.	85%	94%	90%	83%	92%	96%	100%
Alternatives are free from clues	69%	72%	82%	81%	93%	77%	81%

* Rev: total number of reviewers.

^μ^: N: total sample of questions per domain.

^¥^ N/A: Non-applicable.

*Proposed changes to the questions’ text and answers*. In addition to the issues listed in Table 4, ATC members identified several concerns in the items such as 1) the misplacement of questions in the domains; 2) the repetition of questions; 3) the absence of person-first language; and 4) inconsistency in the use of terms. Considering these problems and the performance of questions and domains, the ATC decided to review all 145 items (both flagged and unflagged questions) that comprised the ISWP Basic Test item pool.

#### Phase 2 & 3: Team synchronous meeting and pre-final version

A total of 10 two-hour meetings were held between ATC teams (n = 7) to review all questions (stem and answer options), their domain allocation, and to prepare the final set of questions for content-validity analysis. Members used the results from the qualitative review to guide the revision process. This procedure resulted in dropping 34 items (23.4%), updating 111 items (76.5%) and creating 61 new items. Table 5 presents the review details grouped by domain. The item revision involved updating the content and terminology considering evidence-based materials, complying with the questions guidelines and copy editing (grammar, flow, spelling, and punctuation). In addition, ATC members deleted duplicated questions and re-categorized questions when they were in the wrong domain.

**Table 5:** Question analysis grouped by domain.

Domain	N	Dropped	Revised	New Items Added
Assessment	34	7	27	16
Prescription	17	4	13	11
Fitting	22	3	19	7
Production	12	4	8	3
Training	26	5	21	22
Process	25	9	16	0
Follow-up	9	2	7	2
**Total, n (%)**	145	34 (23.4)	111 (76.5)	61

#### Phase 4. Classification and final revision

A total of 172 multi-choice questions were re-classified considering the WHO 8-steps and retained for content validity assessment. Table 6 includes the new proposed items’ domain classification, the sub-domain definitions, and the number of questions per domain. The ATC decided to use the same sub-domains comprised in the WHO Basic Package with two caveats. One, due to the limited information provided in the manual regarding *step 4*: *funding and ordering*, and the variability of funding and ordering processes in international settings, this step was not included as a sub-domain to test basic manual wheelchair-service-provision knowledge. And two: *step 3*: *prescription and step 5*: *product (wheelchair) preparation* were combined in one sub-domain because the two steps were considered synergistic.

**Table 6:** Questions and domain classification for content validity.

Domain: Basic manual wheelchair-service-provision knowledge		
Sub-domains	Definition	N
**Core Knowledge**	General information about best practices to undertake the wheelchair-service-provision process.	8
**Assessment**	Refers to step 2 of the WHO 8 steps that includes the assessment interview and the physical assessment of the wheelchair user. The information collected in the interview includes the wheelchair user’s contact information, physical condition, lifestyle and environment, and information about the existing wheelchair (if applicable). In the physical assessment, the provider identifies the presence, risk, or history of pressure injuries; identifies the user’s method of pushing; obtains the user’s body measurements that will help select the size of the wheelchair; and assesses current wheelchair skills	43
**Prescription and Product Preparation**	Refers to steps 3 (prescription) and 5 (product preparation) of the WHO 8 steps. The prescription includes the selection of the best available wheelchair and cushion for the user based on his/hers needs; the size of the wheelchair and cushion (considering the body measurements obtained in step 2); and the features (characteristics) of the wheelchair based on the user’s needs. The product preparation includes wheelchair adjustments and revisions made by the provider to ensure that the wheelchair is safe and ready to be used.	38
**Fitting**	Refers to step 6 of the WHO 8 steps. The fitting includes reviewing that the wheelchair size and adjustments, the pressure, the posture, and the fit of the wheelchair user while moving are adequate; and the cushion is working properly.	29
**User Training**	Refers to step 7 of the WHO 8 steps. The user training includes teaching the wheelchair user and (if appropriate, the caregiver) wheelchair skills, transfers, prevention of pressure injuries, and cushion and wheelchair maintenance tasks.	41
**Follow-up**	Refers to step 8 of the WHO 8 steps. In the follow-up, the wheelchair provider checks the condition of the wheelchair, its fit, the need for additional training, and that the wheelchair still meets the user’s needs.	13
	**Total**	172

### Objective three: Evaluate the content validity of the items included in ISWP Basic Test Version 2

Eight subject matter experts completed the survey. Their characteristics are documented in Table 7. The group had a gender-balanced representation with a mean age of about 40 years old and an average of 18 years involved in the wheelchair sector. Most members’ primary occupation was clinically oriented with a focus on wheelchair-service-provision.

**Table 7:** Characteristics of members of the subject matter expert group.

Characteristics	Subject Matter Experts Group (n = 8)
**Gender, n (%)**	
Female	4 (50)
Male	4 (50)
**Age, mean (SD)** *	39.6 (10.89)^μ^
**Country of origin, n (%)**	
Canada	2 (25)
Colombia	1 (12.5)
India	3 (37.5)
Ireland	1 (12.5)
United States of America	1 (12.5)
Primary language, n, (%)*	
English	4 (50)
Spanish	1 (12.5)
Hindi	1 (12.5)
Tamil	1 (12.5)
Tegulu	1 (12.5)
**Speak other language, n (%)**	5 (62.5)
**Educational level, n (%)**	
4-year degree/bachelor’s degree	4 (50)
Graduate degree/masters’ level	1 (12.5)
Graduate degree/MD, PhD	3 (37.5)
**Profession, n (%)**	
Occupational Therapist	4 (50)
Physical Therapist	4 (50)
**Primary Occupation, n (%)**	
Consultant	1 (12.5)
Professor	2 (25)
Researcher	1 (12.5)
Clinician/Wheelchair Service Provider	3 (37.5)
Other: Director Education and training	1 (12.5)
**Employment status, full-time, n (%)**	8 (100)
**Years involved in wheelchair sector, mean (SD)**	17.8 (11.65)
**Years of wheelchair-service-provision experience as, mean (SD)**	
Clinician	15 (12.75)
Researcher	8 (7.23)^μ^
Educator	16.28 (11.57)^μ^
Leadership in prof organizations	6.86 (4.74)^μ^
**Have taken the ISWP Basic Test: yes, n (%)**	7 (87.5)
**Confidence in knowledge of the WHO WSTP-Basic level, n (%)**	
Neither competent nor incompetent	2 (25)
Somewhat competent	2 (25)
Extremely competent	4 (50)
**Confidence in knowledge of the Wheelchair Skills Program, n (%)**	
Neither competent nor incompetent	1 (12.5)
Somewhat competent	3 (37.5)
Extremely competent	4 (50)

* SD: Standard deviation

^μ^: N = 7

The I-CVI for the 172 multi-choice questions that inquired about *item clarity* ranged from 0.38–1.00, with 66 items (38.4%) falling below the acceptable 78% threshold. In terms of *item relevance*, I-CVIs ranged from 0.57 to 1.00, with 4 items (2.3%) falling below the acceptable 78% threshold; these items did not meet the threshold in clarity either. Seven questions had <80% of agreement about their domain allocation. A good agreement between subject matter experts was found; the S-CVI calculated using the I-CVIs related to item clarity was 84% while using the I-CVIs related to item relevance was 98%. Table 8 groups the content validity results by subdomains.

**Table 8:** Content validity results grouped by domain.

Subdomain	N	I-CVI ≤ 0.78		Dropped	Domain
		Clarity	Relevance		<80%
Core Knowledge	8	7	0	0	0
Assessment	43	27	3	3	6
Prescription and Product Preparation	38	16	0	0	0
Fitting	29	10	1	1	0
User Training	41	3	0	0	1
Follow-up	13	3	0	0	0
**Total, n (%)**	172	66 (38.4)	4 (2.3)	4 (2.3)	7 (4.1%)
**S-CVI**		0.84	0.98		

ATC members reviewed and updated the items’ flagged for clarity review and domain allocation using the feedback provided in the open-ended comments. The 4-items that did not meet the clarity and relevance threshold were dropped, leaving a total pool of 168 items.

## Discussion

In this project, we reviewed and evaluated the psychometric properties of the ISWP Basic Test Version 1 in English, updated the test using a systematic approach guided by stakeholders to develop the ISWP Basic Test Version 2 and evaluated the content validity of this new version. The results from the revision indicate that only 20% of the questions included in the ISWP Basic Test Version 1 in English met the thresholds for difficulty and the index of discrimination considered in the literature as standard levels for effective testing [28, 29]. The ISWP Basic Test Version 2 was developed considering the question and domain performance of Version 1 and guidelines for the development of question texts and answers that resulted in a test pool of 172 new items grouped in six sub-domains. Results from the content validity assessment showed a good agreement related to the items’ clarity and relevance. The feedback provided by reviewers improved the items’ clarity and relevance and retained 168 items for future pilot testing.

### ISWP Basic Test Version 1: Psychometric properties

More than two-thirds of test-takers passed the test in their first attempt of the ISWP Basic Test Version 1 in English. This passing rate differs from the common perception of the need for more wheelchair-service-provision training worldwide [30] and the robust evidence reporting limited competency and education among entry-to-practice rehabilitation students [7, 8, 31] and clinicians [2, 6, 9, 32]. It could be assumed that the high passing rates reflect a homogeneous and highly educated group on wheelchair-service-provision. However, the socio-demographic characteristics of the sample show a diverse group of participants. The sample was represented by test-takers from different continents with various educational levels that ranged from high school to doctorate degrees. Although most test-takers held a bachelor’s degree in rehabilitation professions primarily responsible for wheelchair service delivery, it cannot be expected that their wheelchair-service-provision competence was due to their professional training. Evidence has emerged demonstrating that professional rehabilitation programs worldwide (i.e., Physical Therapy, Occupational Therapy and Prosthetics and Orthotics) have very limited wheelchair-related education in their curricula [33–35]. As such, the passing rates may be associated with other wheelchair training. Unfortunately, the socio-demographic questionnaire did not explore further the characteristics of the training received. In the future, additional information can be obtained regarding test-takers previous wheelchair training.

Based on the results of psychometric properties of the ISWP Basic Test Version 1 and the qualitative review of the test questions conducted in this project, we believe that the high pass rates do not necessarily represent test-takers competency in basic wheelchair-service-provision. Rather, they may represent problems with the test. None of the domains of the ISWP Basic Test Version 1 had the minimum internal consistency to presume a strong relationship between items. The negative KR-20 values in *Fitting*, *Production* and *Follow-up* presumed that one or more of the items in those domains are not performing properly. This may be due to the displacement of questions in the domains. Overall, the results from the internal consistency reliability suggest that the items grouped in each domain are not measuring what they intend to measure. Instead, they are measuring many unknown factors. At the item-level analysis, the results show that 80% of the total test pool did not meet the thresholds for item difficulty and index of discrimination suggested in the literature [16, 28]. Most questions exceeded the recommended difficulty threshold for multiple-choice questions, indicating that more than two-thirds of the questions could be responded to intuitively without adequate knowledge of wheelchair-service-provision. Similarly, about two-thirds of the questions were unable to distinguish between test-takers who are knowledgeable in the content area and those who are not [16]. These results suggest that the ISWP Basic Test Version 1, which has been used as part of an international wheelchair-service-provision certification process, does not discriminate between test-takers with basic knowledge of wheelchair-service-provision and those without it. Our results support the Rushton et al [14] assumption that the ISWP Basic Test may not be accurately measuring basic wheelchair-service-provision competency, and that the low degree of internal consistency between the translated French-Canadian version and the original version may be due to issues with the original test.

### Development of the ISWP Basic Test Version 2

Sixty-one new items were developed for ISWP Basic Test Version 2, the largest number of which (n = 22) corresponds to the *User training* sub-domain. This was made possible by using the WSP, a gold standard training package, as a reference [4]. The questions added relate to indoor wheelchair skills, community skills, advanced skills and motor learning principles. Most of this content is not covered by the WHO Basic Package, but it is in the WSP manuals and training resources that are freely available online. We placed particular emphasis on increasing the pool of *User training* questions, as recent evidence reveals that 1) few professionals provide wheelchair skills training to their clients and caregivers [36] and 2) rehabilitation professionals receive limited to no training on wheelchair skills [35, 37], despite the fact that such training has been found to be highly effective [38, 39]. We hope that increasing the pool of this sub-domain draws attention to educators, trainers, and trainees on the importance of wheelchair skills training as part of the wheelchair service delivery process.

In the ISWP Basic Test Version 2, we have included the domains Core Knowledge and six of the WHO 8-steps (i.e., *Assessment*, *Prescription*, *Product preparation*, *Fitting*, *User training*, and *Follow-up*). The domain Core Knowledge was populated with many items previously grouped in the sub-domain *Process*. According to Gartz et al., the sub-domain ‘*Process*’ was created in the development of the ISWP Basic Test Version 1 with content from two of the WHO-8 steps., *Referral and appointment* and *Funding and ordering* [5]. However, the quality review of the Test’s questions’ text and answers revealed that many questions were misplaced in the domains and some, like those included in *Process*, did not correspond to the titled content. These findings increased the scope of the project and resulted in all questions being re-classified.

In terms of the WHO 8-steps, we decided not to include the steps of *Referral and appointment* and *Funding and ordering* because they lack sufficient content to create an adequate pool of questions. Without an adequate sample size per sub-domain, it is difficult to create items with a range of difficulty levels that allow to discriminate between test takers’ knowledge [40]. Further, the two domains may be highly influenced by the context [1] and less pertinent to standardize in an international test. That being said, we recognize the importance of these steps and suggest reviewing the decision in the future using the data analysis from subsequent test results. Also, the WHO is in the process of revising its Guidelines and the classification of the components of wheelchair service delivery is likely to evolve based on experience and evidence acquired since its introduction in 2008 [12].

The proposed new classification of sub-domains of the ISWP Basic Test Version 2 is more aligned with the content of the WHO Basic Package. This classification recognizes that basic wheelchair-service-provision knowledge is the combination of two constructs: 1) Core Knowledge, which includes the background knowledge to undertake an appropriate wheelchair service delivery; and 2) Wheelchair Service Steps, the interconnected actions to provide an appropriate wheelchair service delivery [41]. We were particularly interested in keeping Core Knowledge as a domain because, in many places worldwide, wheelchair-service-provision is led by personnel without a healthcare professional degree (e.g., community-based workers, technicians, and local craftsmen) that may benefit from reviewing and assessing this section of the WHO Basic Package [3, 41].

The developed set of questions had an excellent scale content validity index, presuming “strong conceptualizations of constructs, good items, judiciously selected experts, and clear instructions to experts regarding the underlying constructs and rating task” [24]. We received feedback on the clarity of 66 items (38.4%) via open-ended comments that guided the improvement of the questions’ text. Only 4 (2.3%) items were deemed not relevant and were dropped from the final set. The proposed new classification of domains and items was well accepted by our group of subject matter experts who considered that only 7 (4.1%) items did not correspond to the proposed sub-domain.

### Study limitations

A potential limitation of this study is that the feedback received from members of the ATC in the review and update of the items and the SMEG during the content-validity analysis is likely to include bias in their expertise and experience. We used a purposive and convenience sampling method to recruit members from both groups respectively and the groups may have not had experts from all steps of the wheelchair service delivery process.

### Future directions

The next phase of this project includes 1) pilot test the questions, 2) evaluate the psychometric properties (i.e., item difficulty, item discrimination index, and domains’ internal consistency reliability), 3) select the final question pool, and 3) determine the domains’ weighting for the new ISWP Basic Test Version 2. Once the second phase of the project is complete, the ISWP Basic Test Version 2 will replace Version 1 and will be available to the general public. It is important to note that the ISWP Basic Test Version 2 is not parallel in form with Version 1. The questions included in the newest version have significantly changed, such that there was not a single question that was un-edited between versions and new questions were developed considering other training materials As such, the test sub-domains should not be compared across versions unless test equating is conducted [42].

ISWP is encouraged to allocate funding to complete this project promptly to offer a psychometrically sound test to measure basic wheelchair-service-provision competency. Considering that current and future versions of the Test are part of a fee-based certification process, we deemed it appropriate that stakeholders and subject matter experts be compensated for their time and contributions. Seven years passed between the launch of the ISWP Basic Test Version 1 and the revision of its psychometric properties. We recommend the development of a plan and task force to periodically review and update the Test in line with best practices in psychometric analysis including modern test theory and explore computer adaptive testing to ensure a fair and representative exam while minimizing test taker burden [43].

## Conclusions

Valid and reliable scores from measurement tools for basic manual wheelchair-service-provision are necessary to support competency development and to promote good practice. The ISWP Basic Test is an international test that has been used since 2015 to evaluate basic manual wheelchair-service-provision knowledge. In spite of the study limitations and the need for further study and Test monitoring, this is the first study to explore the psychometric properties and conduct a qualitative review of the ISWP Basic Test Version 1. The results from the psychometric study revealed that the current version of the test lacks the minimum standard parameters for effective testing. The new items developed by experts in the field have shown excellent content validity. Future work will pilot test the items and determine the weighting of the subdomain for the new ISWP Basic Test Version 2.

## Supporting information

S1 Table Questions’ guidelines table. (DOCX)

S2 Table Domain feedback form. (DOCX)

S1 Dataset (XLSX)
