# Supplementary material for: Psychometric properties of the International Society of Wheelchair Professionals’ basic manual wheelchair-service-provision knowledge Test Version 1 and development of Version 2
Source: PLoS One. 2023 Mar 23;18(3):e0281584. doi: 10.1371/journal.pone.0281584 (PMC10035907; doi:10.1371/journal.pone.0281584)
Supplement: S1 Table — (DOCX) [file pone.0281584.s001.docx]

**S1. Questions’ Guidelines Table**

| **Guidelines** | **Score: Y/N/NA*** |
| --- | --- |
| **Question Text** |  |
| Stem is meaningful by itself and presents a definite problem. |  |
| Stem does not contain irrelevant material. |  |
| Stem is a question or partial sentence. |  |
| Stem is negatively stated only when significant learning outcomes require it. Negative statement is in italics or capitalization. |  |
| Stem targets a specific cognitive process. |  |
| **Answers’ options** |  |
| All alternatives are plausible. |  |
| Alternatives are stated clearly and concisely. |  |
| Alternatives are mutually exclusive. |  |
| Alternatives are homogenous in content. |  |
| Alternatives are free from clues. |  |

* Y=yes, N=no, NA= not applicable.
