# Supplementary material for: Psychometric properties of the International Society of Wheelchair Professionals’ basic manual wheelchair-service-provision knowledge Test Version 1 and development of Version 2
Source: PLoS One. 2023 Mar 23;18(3):e0281584. doi: 10.1371/journal.pone.0281584 (PMC10035907; doi:10.1371/journal.pone.0281584)
Supplement: S2 Table — (DOCX) [file pone.0281584.s002.docx]

**S2. Domain Feedback Form (Example)**

Section 1. Domains’ summary table.

| **Domain** | **Prescription** |
| --- | --- |
| Pool of Questions, n |  |
| Flagged for revision, n (%) |  |
| Reliability (KR-20) |  |

Section 2 & 3. Flagged and Unflagged questions for revision.

| **Cd** | **Question Text** | **ID (*P*)** | **IDI** | **Answers’ option/ text** | | **N (SD)** |
| --- | --- | --- | --- | --- | --- | --- |
| **501394** | Stem |  |  | A |  |  |
|  |  |  |  | B |  |  |
|  |  |  |  | C |  |  |
|  |  |  |  | D |  |  |
| **Feedback** | Proposed changes to stem | | | A | Proposed changes to answer options | |
|  |  |  |  | B |  |  |
|  |  |  |  | C |  |  |
|  |  |  |  | D |  |  |
|  | Additional feedback | | | | | |
|  |  |  |  |  |  |  |
|  |  |  |  |  |  |  |
|  |  |  |  |  |  |  |

Cd: Question’s code; ID (*P*): Item difficulty; IDI: Item discrimination index; N: Sample of questions; SD: Standard deviation.
